# Core Bioactive Components Promoting Blood Circulation in the Traditional Chinese Medicine Compound Xueshuantong Capsule (CXC) Based on the Relevance Analysis between Chemical HPLC Fingerprint and *In Vivo* Biological Effects

**DOI:** 10.1371/journal.pone.0112675

**Published:** 2014-11-14

**Authors:** Hong Liu, Jie-ping Liang, Pei-bo Li, Wei Peng, Yao-yao Peng, Gao-min Zhang, Cheng-shi Xie, Chao-feng Long, Wei-wei Su

**Affiliations:** 1 Guangzhou Quality R & D Center of Traditional Chinese Medicine, Guangdong Key Laboratory of Plant Resources, School of Life Sciences, Sun Yat-sen University, Guangzhou, P.R. China; 2 Guangdong Zhongsheng Pharmaceutical Co., Ltd., Dongguan, P.R. China; Heidelberg University, Germany

## Abstract

Compound xueshuantong capsule (CXC) is an oral traditional Chinese herbal formula (CHF) comprised of *Panax notoginseng* (PN), *Radix astragali* (RA), *Salvia miltiorrhizae* (SM), and *Radix scrophulariaceae* (RS). The present investigation was designed to explore the core bioactive components promoting blood circulation in CXC using high-performance liquid chromatography (HPLC) and animal studies. CXC samples were prepared with different proportions of the 4 herbs according to a four-factor, nine-level uniform design. CXC samples were assessed with HPLC, which identified 21 components. For the animal experiments, rats were soaked in ice water during the time interval between two adrenaline hydrochloride injections to reduce blood circulation. We assessed whole-blood viscosity (WBV), erythrocyte aggregation and red corpuscle electrophoresis indices (EAI and RCEI, respectively), plasma viscosity (PV), maximum platelet aggregation rate (MPAR), activated partial thromboplastin time (APTT), and prothrombin time (PT). Based on the hypothesis that CXC sample effects varied with differences in components, we performed grey relational analysis (GRA), principal component analysis (PCA), ridge regression (RR), and radial basis function (RBF) to evaluate the contribution of each identified component. Our results indicate that panaxytriol, ginsenoside Rb_1_, angoroside C, protocatechualdehyde, ginsenoside Rd, and calycosin-7-O-β-D-glucoside are the core bioactive components, and that they might play different roles in the alleviation of circulation dysfunction. Panaxytriol and ginsenoside Rb_1_ had close relevance to red blood cell (RBC) aggregation, angoroside C was related to platelet aggregation, protocatechualdehyde was involved in intrinsic clotting activity, ginsenoside Rd affected RBC deformability and plasma proteins, and calycosin-7-O-β-D-glucoside influenced extrinsic clotting activity. This study indicates that angoroside C, calycosin-7-O-β-D-glucoside, panaxytriol, and protocatechualdehyde may have novel therapeutic uses.

## Introduction

Traditional Chinese medicine (TCM) employs compounds called Chinese herbal formulas (CHFs). In the past three decades, these compounds have been attracting increasing attention for their complementary therapeutic effects to western medicines [1], [2]. CHFs are comprised of multiple components and affect numerous targets, yet the bioactive components of most CHFs have not been elucidated. In the Pharmacopoeia of the People's Republic of China (2010), only a few components, or even a single component, based on their content in herbs instead of their clinical activities, were controlled in current CHF quality standards. In order to better guarantee the clinical safety and effectiveness of CHF, the bioactive components evidenced by suitable methods should be controlled.

Bioactivity control, which reflects information directly related to safety and effectiveness, is indispensable in CHF quality control [3]. Therefore, a change from traditional evaluation methods that emphasize chemical characteristics to a method that quantifies biological effects is urgently required [4]. Also, the Guidance for Industry Botanical Drug Products from the U.S. Food & Drug Administration (FDA) (2004) clearly indicated that the functional study of known components in herbal medicine should be improved. The recognition and control of bioactive components has thus become a key technology of CHF development.

According to the theory of modern chemical biology, drug effects are achieved by the regulation of biomacromolecules by small molecules in it. Hence, there must be a group of bioactive components responsible for a drug's clinical effects. The concept of relevance between drug components and effects was first introduced in 2002 [5], and the earliest methodology to match drug effects with prominent peaks in high-performance liquid chromatography (HPLC) fingerprints was reported in 2000 [6]. Kong et al. studied the relationship between the HPLC fingerprint and the antibacterial effects of artificial Calculus bovis with the help of chemometric methods [7]. Lu et al. examined the corresponding relationship between the HPLC fingerprint and the effects of Houttuynia cordata injection using multivariate statistical analysis [8]. In the past decade, studies on the relevance between drug components and effects have provided valuable information to improve TCM development, including quality control, classification, CHF prescription analysis and optimization, the identification of new medicinal plant sources, and drug design.

Compound xueshuantong capsule (CXC) first appeared on the Chinese market in 1996; it is an oral TCM composed of *Panax notoginseng* [PN; root of *Panax notoginseng* (Burk.) F. H. Chen], *Radix astragali* [RA; root of *Astragalus membranaceus* (Fisch.) Bge. var. *mongholicus* (Bge.) Hsiao], *Salvia miltiorrhizae* (SM; root of *Salvia miltiorrhiza* Bge.), and *Radix scrophulariaceae* (RS; root of *Scrophularia ningpoensis* Hemsl.) in a 25∶8∶5∶8 ratio.

The concept of blood stasis is described in TCM theory as a slowing or pooling of the blood due to disruption of the heart's Qi [9]. It is often understood in biomedical terms as hematological disorders, such as hemorrhage, congestion, thrombosis, and local ischemia [9]. In 1998, Mchedlishvili et al reported that decreased blood flow velocity disturbs normal blood flow, which indicates hemorheological abnormalities [10]. It was explained by pathology as a state resulting from a sluggish or impeded blood flow in the body, or abnormal blood outside the vessels that remains in the body and fails to disperse [11]. Once blood stasis develops, the circulation will be further affected, resulting in new pathological changes [11], [12].

**Figure 1:**
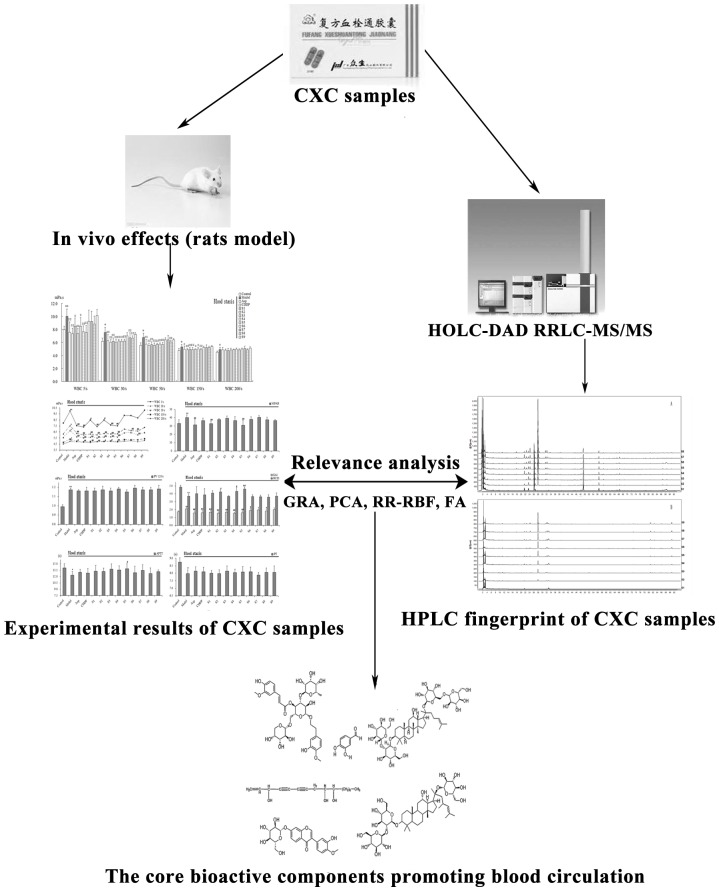
The research process for finding the bioactive components promoting blood circulation in CXC.

The core bioactive components of CXC that improve blood circulation impairment remain unknown. The present study was designed to assess the relationship between CXC components and circulatory effects and the specific research process was shown in Fig. 1.

## Materials and Methods

### 1 Ethics statement

We obtained 130 specific-pathogen free (SPF) male Sprague–Dawley rats, weighing 180–220 g, from the Guangdong medical experimental animal center (certification No: SCXK-(Yue) 2008-0002) and raised in an SPF laboratory (license: SCXK-(Yue) 2009-0020) at Sun Yat-sen University. This experiment was approved by Animal Ethics Committee of the Life Sciences School, Sun Yat-sen University (permission No: 2012082502). The temperature of the SPF laboratory was 20–23°C, and the relative humidity was 50–65%. Rats were fed with standard pellet feed. Experiments began after the rats had adapted to the new environment for one week. The harm to rats was minimized during the experimental process.

### 2 CXC sample preparation

Based on the original formula, CXC samples were prepared by changing the content of 4 herbs. The specific weights of 4 herbs were designed according to a four-factor, nine-level uniform design (UD) (Table 1). For each CXC sample, PN was soaked in 50% ethanol twice. The soaking liquids were filtered separately, and the two filtrates were mixed together. The ethanol was recovered, and the water was evaporated to produce the PN extraction. Then RA, SM, and RS were mixed together and refluxed twice with 50% ethanol. The reflux extraction liquid was filtered separately, and both filtrates were mixed together. The ethanol was recovered, and the water was evaporated to produce the three-herb extraction, which was combined with the PN extraction to form the CXC sample [13]. Each CXC sample was made into the same dose of 152 mg/ml by diluting with normal saline. The 4 herbs (batch No: 120523) were provided by Guangdong Zhongsheng Pharmaceutical Co., Ltd. (Dongguan, China).

**Table 1:** Weights and percentage of 4 herbs in CXC samples under uniform design.

	Extract (g)	PN (g-%)	RA (g-%)	RC (g-%)	SM (g-%)
S1	400	696-64.0	98-9.0	294-27.0	0-0
S2	405	730-61.5	267-22.5	160-13.5	30-2.5
S3	401	771-59.0	471-36.0	0-0	65-5.0
S4	405	579-56.5	46-4.5	323-31.5	77-7.5
S5	400	600-54.0	200-18.0	200-18.0	111-10.0
S6	402	626-51.5	383-31.5	55-4.5	152-12.5
S7	411	474-49.0	0-0	348-36.0	145-15.0
S8	404	486-46.5	141-13.5	235-22.5	183-17.5
S9	402	500-44.0	307-27.0	102-9.0	227-20.0

### 3 Experimental Instruments and reagents

The study used a numerical control ultrasonic cleaning machine (KQ-250DE; Kunshan Ultrasonic Instrument, Suzhou China); 1/100,000 an electronic analytical balance (BP211D; Sartorius, Goettingen, Germany),an Ultimate 3000 DGLC HPLC (Dionex, Sunnyvale, CA, USA), an Agilent HPLC-MS (1200RRLC-6410, triple-quadrupole tandem mass spectrometry; Agilent, Santa Clara, CA, USA), an ultra-low temperature freezer (BCD-568W; Haier), refrigerated centrifuges (5430R, TD5A-WS, TDL-5M; Eppendorf, Hamburg, Germany), a platelet aggregation analyzer (LBY-NJ4; Beijing Precil Instrument Co., Beijing, China), a fully automatic blood coagulation analyzer (CA-510; Sysmex, Kobe, Japan), a fully automatic self-cleaning hemorheology analyzer (LBY-N6B, Beijing Precil Instrument Co.), an automatic dynamic blood sedimentation tester (LBY-XC40, Beijing Precil), and a chromatographic column (Dionex Acclaim 120 C18, 3 µm, 150 mm×4.6 mm).

Adrenalin hydrochloride (Adr) was purchased from Shanghai Harvest Pharmaceutical Co., Ltd. (Shanghai, China; state medical permission No: H31021062, batch No: 20111109). Aspirin (Asp) was purchased from Jilin Luwang Pharmaceutical Co., Ltd. (Jilin, China; state medical permission No: H22025784, batch No: BTA7WH2). Compound danshen dripping pills (CDDP) were purchased from Tianjin Tasly Pharmaceutical Co., Ltd. (Tianjin, China; state medical permission No: Z10950111, batch No: 120201). HPLC solvents were of HPLC grade.

### 4 HPLC and cluster analysis of CXC samples

Liang et al have previously established the standard HPLC fingerprint of CXC and identified 21 components [14]. The HPLC fingerprints of nine CXC samples were obtained using the established method, and a Dionex Acclaim 120 C_18_ column (3 µm, 150 mm×4.6 mm) was used with acetonitrile (A) and 0.05% orthophosphoric acid (B) in gradient elution mode. The elution profile was 0–50 min (15%→34%A), 50–95 min (34%→75%A); the detection wavelengths were 203 nm and 270 nm; the column temperature was set at 25°C, and the flow rate was 1.0 ml/min. Peak areas were calculated with a Chromeleon 6.8 Chromatography Data System (Dionex). The peak areas of 21 identified components were adopted as independent component variables (Table 2). Cluster analysis of the nine CXC samples was conducted in SPSS 18.0. The clustering method was between-groups linkage, and the distance calculating method was Pearson's correlation.

**Table 2:** Identified components in CXC and their attribution.

variables	retention time	attribution	identified component
P1	*11.3	SM	calycosin-7-O-β-D-glucoside
P2	17.8	RA	lithospermic acid
P3	21.6	SM	angoroside C
P4	23.6	RS	notoginsenoside R_1_
P5	*24.3	SM	salvianolic acid A
P6	26.3	PN	ginsenoside Rg_1_
P7	26.8	SM	ginsenoside Re
P8	*28.1	RA	salvianolic acid B
P9	29.5	PN	9,10-dimethoxypterocarpan-3-O-β-D-glucoside
P10	49.5	PN	ginsenoside Rb_1_
P11	56.8	SM	ginsenoside Rd
P12	*80.9	RA	cryptotanshinone
P13	5.6	RA	protocatechualdehyde
P14	*22.6	RS	rosmarinic acid
P15	25.2	PN	Ononin
P16	32	RA	calycosin
P17	33.5	PN	harpagoside
P18	52	SM	formononetin
P19	81.9	PN	panaxytriol
P20	82.7	SM	tanshinoneI
P21	*90.8	SM	tanshinoneII_A_

a) PN-Panax notoginseng, RA-Radix astragali, SM-Salvia miltiorrhizae, RS-Radix scrophulariaceae.

b) * means the characteristic peak could be detected at both 203 nm and 270 nm. For these characteristic peaks, the bigger peak areas were chosen for the calculation.

### 5 Experimental animal model

Rats were divided randomly into 13 groups: control, model, Asp (100 mg/kg/d), CDDP (800 mg/kg/d), and 1–9 CXC samples (1,520 mg/kg/d), with 10 rats in each group. Rats received treatment (10 ml/kg/d) via gavage once daily for 10 consecutive days. The control and model groups received the same volume of normal saline. Thirty minutes after the tenth administration, all rats (except those in the control group) were subcutaneously injected with Adr (0.8 mg/kg), while the control group was subcutaneously injected with the same volume of normal saline. Two hours later, all the rats (except those in the control group) were kept in ice-cold water (0–4°C) for 5 minutes. Two hours later, they received a second subcutaneous injection of Adr (0.8 mg/kg) [15]. After the re-injection, all the rats were fasted for 12 hours and then given the last drug treatment.

### 6 Blood sample collection

Chloral hydrate (10%, 3.5 ml/kg) was administered via intraperitoneal injection for anesthesia. Blood was drawn from the abdominal aorta minutes after the last administration and collected into plastic tubes with 3.8% sodium citrate (citrate/blood: 1/9, v/v). All the blood samples were strictly processed and examined using standard operating procedures.

### 7 Hemorheology and blood clotting activity assessments

Blood plasma obtained from 1.5 ml blood (3,820 r/min, 20°C, 15 min) was put into a Sysmex CA-510 fully automatic blood coagulation analyzer to determine activated partial thromboplastin time (APTT), prothrombin time (PT), and plasma viscosity (PV). Blood (0.9 ml) was put directly into a fully automatic self-cleaning hemorheology analyzer (LBY-N6B) to measure the erythrocyte aggregation index (EAI) and the red corpuscle electrophoresis index (RCEI), as well as whole-blood viscosity (WBV) at 5/s, 30/s, 50/s, 150/s, and 200/s shear rates. Blood (0.9 ml) was centrifuged (3,820 r/min, 20°C, 15 min) for hematocrit detection by an automatic dynamic blood sedimentation tester (LBY-XC40). Platelet-rich plasma (PRP) was separated from 3.0 ml blood sample (500 r/min, 20°C, 10 min), then the remaining without PRP was used to obtain platelet-poor plasma (PPP) (3,000 r/min, 20°C, another 10 min). Platelet counts were kept approximate 200×10^9^/L in each PRP sample. Platelet aggregation was induced by 5 µl adenosine diphosphate (ADP). PRP (300 µl) and PPP (300 µl) were used to detect maximum platelet aggregation rate (MPAR) on a platelet aggregation analyzer (LBY-NJ4). All the tests were completed in the drug non-clinical evaluation research center of Guangzhou General Pharmaceutical Research Institute.

### 8 The relevance analysis between HPLC fingerprint and drug effects: mathematical approaches

The peak areas of 21 identified components were regarded as 21 independent component variables (p1–p21). For each component, the corresponding peak area was divided by its area average in 9 CXC samples for nondimensionalization (Table 3). For each measurement index, the effect of each CXC sample was represented by the average value in the corresponding group. The negative indices (the larger the value, the smaller the effect) were switched to positive by taking the reciprocal. Then, the average value in each group was divided by the corresponding average in 9 CXC sample groups for nondimensionalization. The dimensionless data are shown in Table 4.

**Table 3:** Dimensionless data of peak areas of 21 identified components in CXC samples.

	P1	P2	P3	P4	P5	P6	P7	P8	P9	P10	P11	P12	P13	P14	P15	P16	P17	P18	P19	P20	P21
S1	0.6823	0	1.1093	1.0188	0	1.0449	1.0521	0	0.6690	1.0756	1.0546	0	0.4866	0	0.5149	0.4949	1.4719	0.3970	0.9984	0	0
S2	1.0825	0.3681	0.7195	1.1799	0.2721	1.1563	1.1527	0.2923	1.0180	1.1708	1.1108	0.4045	0.5901	0.2509	1.1926	1.2833	0.7841	1.2195	1.1182	0.3331	0.3030
S3	1.6958	0.6884	0	1.4255	0.6280	1.3704	1.3594	0.6321	2.2067	1.3528	1.3304	0.7441	0.6254	0.6019	2.2000	2.4342	0	2.2279	1.3585	0.6896	0.6634
S4	0.5989	0.7612	1.5049	0.9209	0.6676	0.9401	0.9627	0.7057	0.4466	0.9438	0.9603	0.7468	0.9843	0.6742	0.4537	0	1.7285	0.2086	1.1460	0.8185	0.7278
S5	1.0368	1.0170	1.1083	1.0010	0.9649	1.0288	1.0074	0.9991	0.9325	1.0235	0.9836	1.0626	1.0476	0.9982	0.9362	0.8109	1.0685	1.0928	0.9597	1.1096	1.0627
S6	1.4964	1.2740	0.6151	1.0236	1.3492	1.0605	1.0451	1.3509	1.6056	1.0517	1.0234	1.0784	0.9518	1.3713	1.7208	1.8144	0.3430	1.7545	0.9512	1.2277	1.3627
S7	0	1.3385	1.6965	0.7136	1.3052	0.7182	0.7375	1.3301	0	0.7363	0.7806	1.2388	1.4118	1.3367	0	0	1.7273	0	0.8789	1.3309	1.2368
S8	0.9407	1.5637	1.2157	0.8019	1.6644	0.7984	0.8029	1.5830	0.7142	0.7788	0.8553	1.5853	1.3891	1.6119	0.5496	0.6290	1.1514	0.6057	0.8666	1.7964	1.6648
S9	1.4668	1.9893	1.0307	0.9147	2.1487	0.8824	0.8800	2.1069	1.4073	0.8668	0.9009	2.1395	1.5132	2.1549	1.4322	1.5333	0.7253	1.4938	0.7225	1.6941	1.9787

a) P1–P21 represent 21 identified components and are chosen to be the independent component variables for the calculation.

**Table 4:** Dimensionless data of effects of CXC samples.

	WBV (mPa.s)					PT (s)	APTT	PV120/s	MPAR	EAI	RECI
	5/s	30/s	50/s	150/s	200/s		(s)	(mPa.s)			
S1	0.9960	1.0508	1.0506	1.0283	1.0403	0.9955	0.9909	1.0048	1.1025	1.0479	1.0399
S2	1.1472	1.0724	1.0711	1.0361	1.0139	0.9924	0.9853	1.0005	0.9611	1.1029	1.0754
S3	0.9977	1.0378	1.0404	1.0258	1.0036	1.0127	1.0301	1.0102	0.9283	1.0323	0.9285
S4	1.1058	1.0471	1.0371	1.0164	0.9939	1.0002	1.0161	0.9952	0.9987	1.1274	1.1020
S5	1.1243	1.0447	1.0432	1.0329	1.0188	1.0048	1.0338	1.0146	1.1793	1.0740	1.1708
S6	0.9128	0.9238	0.9289	0.9691	0.9879	1.0079	0.9656	0.9826	0.9565	0.9219	0.9230
S7	0.9182	0.9610	0.9261	0.9726	0.9717	0.9799	1.0469	1.0037	0.9016	0.8877	0.9162
S8	0.9603	0.9704	0.9768	0.9739	1.0083	1.0033	0.9488	0.9994	0.9728	0.9426	0.9048
S9	0.8377	0.8918	0.9258	0.9449	0.9616	1.0033	0.9824	0.9889	0.9991	0.8633	0.9394

Factor analysis (FA): five factors with no mutual correlation were extracted from 11 intercorrelated indices. The relevance between 11 indices and 5 factors was calculated with a rotation matrix method to explain the clinical meaning of the factors (Table 5). The scores of five factors were then calculated to be the new dependent variables F1–F5 (Table 6). The processes above were implemented in SPSS 18.0 (Factor Analysis, Data Reduction, Analyze).

**Table 5:** The relevance between 11 indices and F1–F5.

	F1	F2	F3	F4	F5
EAI	0.96	0.19	0.09	0.08	0.08
WBV 5/s	0.93	0.22	0.14	0.05	−0.06
WBV 30/s	0.90	0.08	0.21	0.35	−0.07
WBV 50/s	0.90	0.16	0.09	0.35	0.10
WBV 150/s	0.87	0.16	0.24	0.36	0.04
RCEI	0.71	0.67	0.18	−0.10	−0.08
MPAR	0.20	0.94	0.04	0.25	0.13
APTT	0.16	0.04	0.96	−0.05	−0.16
PV	0.34	0.17	0.64	0.63	−0.04
WBV 200/s	0.60	0.31	−0.19	0.70	0.05
PT	0.03	0.08	−0.14	0.01	0.99
Variance contribution %	61.61	14.20	8.68	7.25	6.26
Variance contribution Accumulated %	61.61	75.81	84.49	91.74	98.00

a) F1–F5 represents Factor 1–5 respectively.

b) Variance contribution means how much the corresponding factor reflects the original data.

c) The larger the absolute value, the higher the relevance between indices and factors. This table is helpful to reveal the clinical importance of each factor.

**Table 6:** The scores of five factors of CXC samples.

	F1	F2	F3	F4	F5
S1	0.1860	0.8201	−0.6073	1.5533	−0.5988
S2	1.5403	−0.5542	−0.6996	−0.1696	−0.9001
S3	0.3091	−1.4263	1.2073	0.7950	1.6147
S4	1.2594	0.0493	0.1388	−1.4871	0.0288
S5	0.3325	1.9929	1.1165	0.2402	0.4671
S6	−0.6231	−0.2070	−1.1009	−0.8173	0.6750
S7	−1.0138	−0.7583	1.3275	−0.0950	−1.8381
S8	−0.5457	−0.4002	−1.1856	0.9853	0.1786
S9	−1.4446	0.4838	−0.1967	−1.0048	0.3727

a) The scores of F1–F5 are chosen to be the new dependent variables for the calculation.

In grey relational analysis (GRA), the grey relational degree (GRD) is used to rank the influence of compared series, which can be represented by the relative distance between the compared series and reference series in an imaging grey space without making any prior assumptions about the distribution type [16]. In the experiment, five factors (F1–F5) were chosen as 5 reference series, and 21 component variables (p1–p21) were chosen as 21 compared series. Then, the GRD between the compared and reference series was calculated with a resolution ratio of 0.5 [16]. The higher the GRD, the greater the effect of the corresponding component.

For principal component analysis (PCA), 21 intercorrelated original independent component variables were recombined into two mutual independent principal components (Table 7), which were regarded as new independent variables. Then, the regression equations between the two principal components and the factors were constructed by a stepwise regression analysis approach. Once a strict regression equation was established (P<0.05), two principal components would be replaced by the 21 original component variables (Table 7) to form another proper multiple linear regression equation, of which the regression coefficients (RCs) were used to evaluate the contribution of each component. In case of no available equations (P>0.05), the Pearson's correlation coefficients (PCCs) between the 21 original component variables and factors were directly calculated. In particular, for F1 and F5, the available equations were as follows: F1 = 1–0.552× (Component 1) (P<0.05), F5 = 1+0.436× (Component 2) (P<0.05). For F2, F3, and F4, PCCs were calculated to evaluate the contribution of each component. The processes above were implemented in SPSS 18.0 (Factor Analysis, Data Reduction, Analyze; Linear module, Regression, Analyze; Pearson Correlation, Correlations, Analyze).

**Table 7:** The scores of two principal components (extracted from 21 component variables).

	P1	P2	P3	P4	P5	P6	P7	P8	P9	P10	P11	P12	P13	P14	P15	P16	P17	P18	P19	P20	P21
Component 1 60.89%	0.04	−0.05	−0.07	0.08	−0.05	0.08	0.08	−0.05	0.05	0.08	0.08	−0.05	−0.07	−0.05	0.05	0.05	−0.05	0.05	0.06	−0.06	−0.05
Component 2 35.78%	0.10	0.09	−0.07	0.03	0.10	0.02	0.02	0.09	0.10	0.01	0.02	0.10	0.05	0.09	0.10	0.10	−0.10	0.10	−0.03	0.08	0.10

a) 60.89% and 35.78% stand for the percentage of variance contribution and two principal components contribute to 96.67% of the total variance.

b) The scores are used for the transformation between two principal components and 21 component variables.

**Figure 2:**
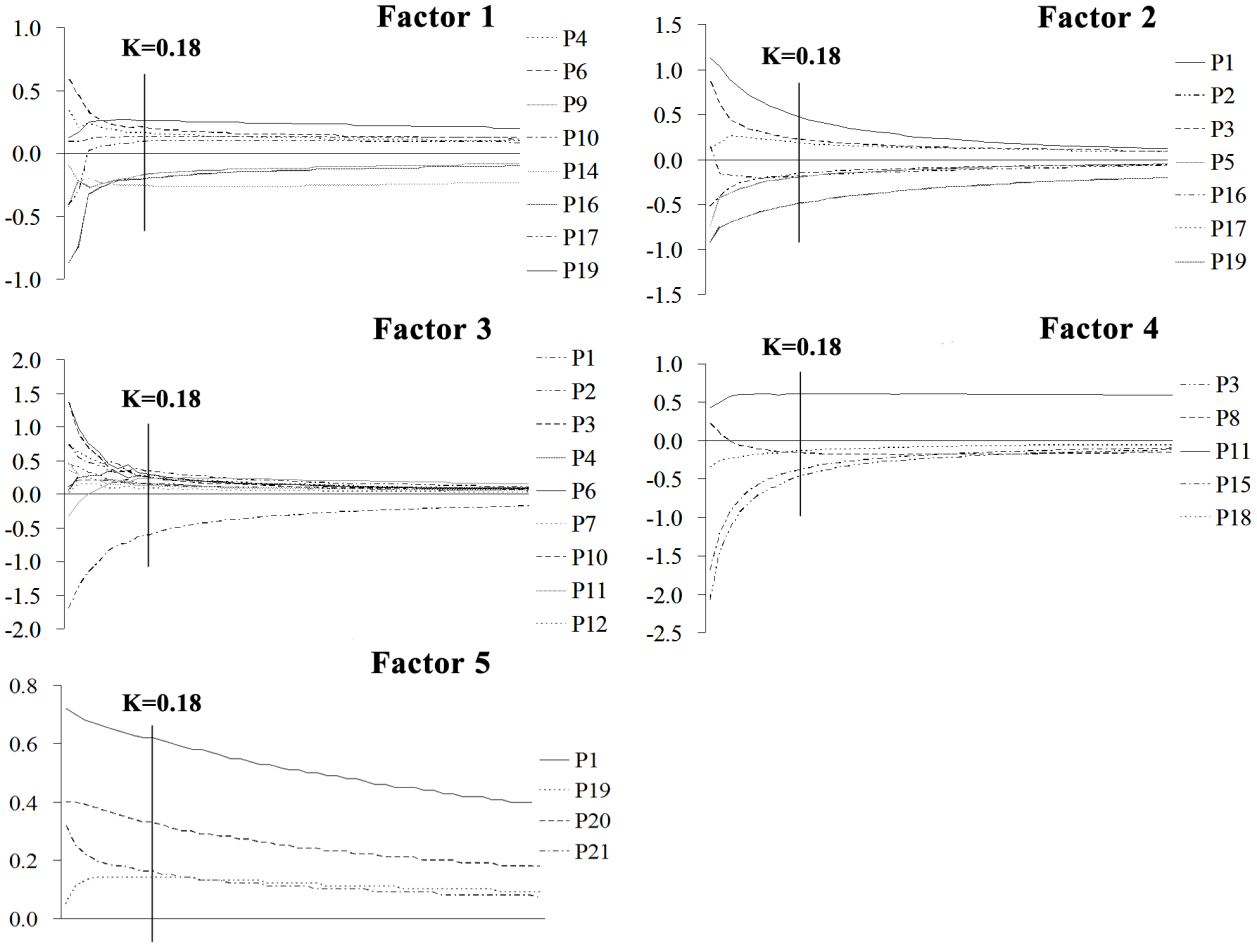
The SRRC ridge trace of selected variables with increase of K for five factors. The minimum K stabilizing all the ridge traces of Factor 1–5 is 0.18.

In ridge regression-radial basis function (RR-RBF) analysis, every ridge parameter K corresponded to a standard ridge regression coefficient (SRRC) for each independent component variable. The ridge trace of each independent component variable, a curve of SSRC on different K (0.02–1, increasing progressively by 0.02), was drawn on a coordinate system. Next, the variables with stable but small absolute values of SRRC and the variables with unstable SRRCs verging infinitely to zero were removed [17]. After that, the remaining variables were used to draw a new ridge trace (Fig. 2), in which the minimum K stabilizing all the ridge traces of independent component variables could be identified. Finally, with this minimum K (0.18), the proper SRRCs of the remaining variables were obtained to evaluate the contribution of each component. Meanwhile, the RBF network between the same remaining variables and each factor (F1–F5) was constructed. Then, the bond value (BV) and standard importance of variable (SIV) of the remaining variables were calculated. BV was used to evaluate the contributions of the components, and SIV represented the reliability of independent component variables in the established RBF network. All the processes above were implemented in SPSS 18.0 (Ridge, Catreg, Regression, Analyze; RBF, Neural Networks, Analyze).

### 9 Statistical analysis

The data are presented as mean ± SD and were evaluated by one-way analysis of variance (ANOVA), Student's *t*-tests, and Dunnett's multiple comparisons in SPSS 18.0 (SPSS Inc., Chicago, IL, USA). P-values less than 0.05 or 0.01 were considered statistically significant.

## Results and Discussion

### 1 CXC sample generation

UD was first proposed by Fang in 1978 and is based on either a quasi-Monte Carlo method or a number-theoretic method. UD is capable of producing samples with high representativeness in the studied experimental domain [18]. Most notably, four-factor, nine-level UD was adopted to guarantee the component difference within 9 CXC samples while reducing workload.

### 2 HPLC and cluster analyses of CXC samples

**Figure 3:**
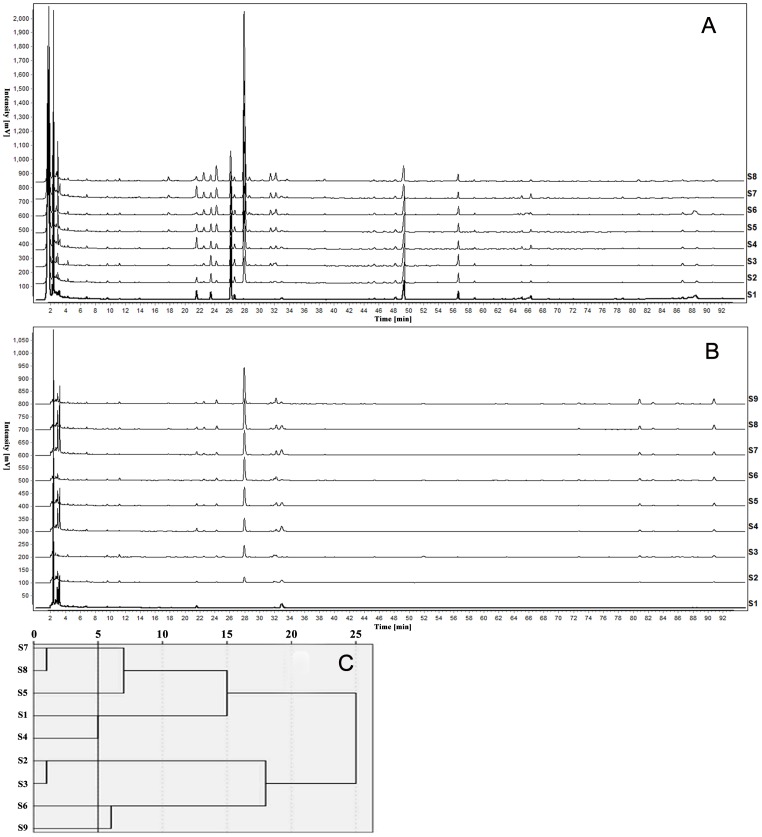
The HPLC fingerprint and cluster analysis results of CXC samples. A, B respectively represents the HPLC fingerprint of 9 CXC samples at wavelength 203 nm and 270 nm, in which 21 components were identified as shown in Table 2. C shows the cluster analysis result of 9 CXC samples.

The HPLC fingerprint of CXC was established in 2012 by our team; we detected 42 common peaks covering 4 herbs, and 21 unique components were identified by RRLC/MS/MS and diode array detector (DAD) spectra (Table 2) [14]. In this experiment, the HPLC fingerprint of 9 CXC samples was constructed as shown in Fig. 3. According to the peak areas of 21 identified components, 9 CXC samples could be divided into 7 categories: S2 and S3 belonged to a class, S7 and S8 belonged to a class, and the rest of the samples each represented a class.

### 3 CXC sample effects in vivo

As an acute stressor, Adr injections can cause various hemorheological disorders, including blood hypercoagulability and increased blood clotting activity [19]. In addition, skin blood flow reduces rapidly when humans are exposed to ice-cold water [20]. It was previously reported that the combination of Adr injection and exposure to ice-cold water could induce poor blood circulation [21]. Therefore, in this experiment, the rats were placed in ice-cold water during the time interval between Adr injections being administered to generate an appropriate model.

Rats with blood stasis exhibit sluggish circulation, leading to changes in hemorrheologic parameters [12]. In recent years, hemorheology has commonly been used to diagnose and prevent diseases and to evaluate the therapeutic effects of drugs on blood circulation [22]. WBV reflects the intrinsic resistance of blood flow in the vasculature, and abnormal WBV is associated with increased cardiovascular risk. The reduction of blood fluidity can lead to tissue ischemia more rapidly in patients with atherosclerotic diseases [23]. WBV at a low shear rate reflects red blood cell (RBC) aggregation and WBV at a high shear rate reflects RBC deformability [24]. WBV is determined not only by RBCs but also by plasma viscosity (PV), which depends on the type and concentration of plasma proteins. Both EAI and RCEI reflect the extent of aggregation among RBCs. Blood platelet aggregation induced by ADP plays a major role in the development and extension of arterial thrombosis [25]. APTT is a test of the intrinsic clotting activity and PT is used to evaluate the overall efficiency of the extrinsic clotting pathway [26]. A prolonged APPT or PT indicates a deficiency in coagulation factors or the presence of inhibitors of coagulation [27].

**Figure 4:**
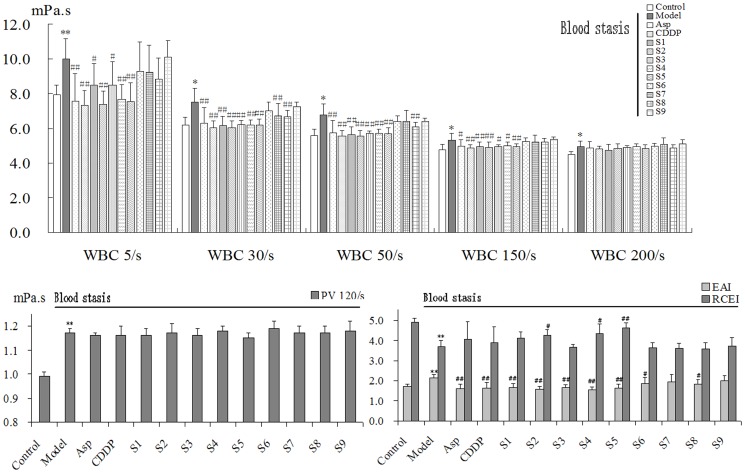
The animal experiment results of CXC samples 1–9. WBV at all shear rates, EAI, and PV increased significantly and RCEI decreased significantly in model rats. After treatment, Asp was significantly effective in decreasing WBV, EAI, and MPAR. CDDP was significantly effective in reducing WBV and EAI. For CXC samples, WBV decreased significantly at 5/s, 30/s, 50/s and 150/s in S1–S5 group. EAI decreased significantly in all groups except for S7 and S9 group. RCEI increased significantly in S2, S4, and S5 group. PV decreased in S1, S3, and S5 group. ^*^ *P*<0.05 and ^**^ *P*<0.01 vs control group, ^#^ *P*<0.05 and ^##^ *P*<0.01 vs model group, n = 10.

**Figure 5:**
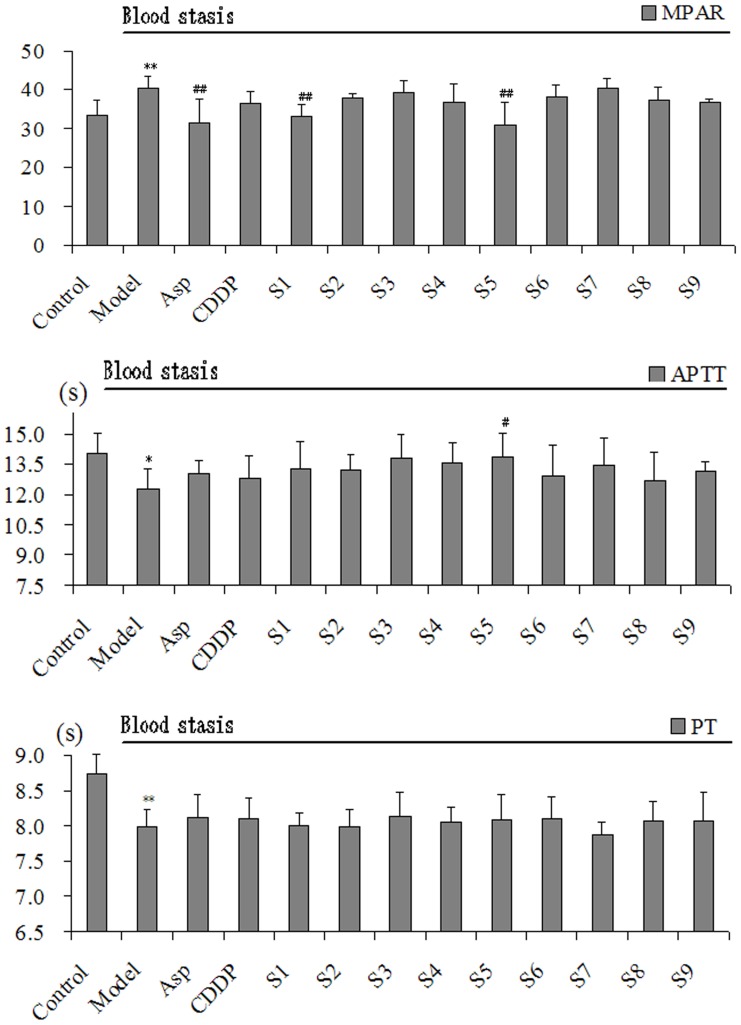
The animal experiment results of CXC samples 1–9. PT and APTT decreased significantly and MPAR increased significantly in model rats. After treatment, APTT was significantly influenced in S5 group. MPAR decreased significantly in S1 and S5 group. PT was prolonged in S3, S5, and S6 group. ^*^ *P*<0.05 and ^**^ *P*<0.01 vs control group, ^#^ *P*<0.05 and ^##^ *P*<0.01 vs model group, n = 10.

Fig. 4 and Fig. 5 show the different effects observed in all 13 treatment groups, with particularly noticeable differences in the 9 CXC groups. There were significant differences between the control rats and model rats for all 11 indices. After treatment, the results showed that indices differed greatly among the 9 CXC samples, and we plan to assess possible reasons for this in subsequent analyses. In addition, the original data that were used to generate Fig. 4 and Fig. 5 were uploaded as Table S1.

### 4 Five factors (F1–F5) extracted from the original data

FA simplifies multivariate data consisting of a large number of intercorrelated variables by grouping them into a smaller set of independent factors or clusters according to the basic underlying relationships between them. Factors are fewer in number than the original variables, account for a significant proportion of data variance, and are useful in predictive regression models [28]. The results in Table 5 show that five mutually independent factors reflected 98% of the original data. Factors 1–5 represented five aspects of the vascular system with clear clinical significance: RBC aggregation (F1), platelet aggregation (F2), intrinsic clotting activity (F3), RBC deformability and plasma proteins (F4), and extrinsic clotting activity (F5).

### 5 Selection and combination of suitable mathematical approaches

Every method has a different theoretical basis and suitable scope, hence a combination of different methods is necessary for a comprehensive and accurate interpretation of the relationship between HPLC data and experimental data. In fact, regarding 21 components from 4 herbs as independent variables probably led to collinearity. Therefore, PCA, which is often used for data reduction by structuring many variables into a much smaller number of components to eliminate variable collinearity, was chosen for this study. Two principal components contributing to 96.67% of the total variance were extracted, and the scores between them and the 21 component variables were calculated for the transformation (Table 6). Ngo et al. demonstrated that RR is clearly superior in that it provides estimated parameters much closer to the true values than those obtained by the ordinary least squares regression method [29]. The RBF network, a powerful type of artificial neural network for forecasting and modeling a multivariable system, is one of the artificial learning methods that describe complex linear or non-linear relationships between inputs and outputs [30]. GRA is suitable for solving problems with complicated interrelationships between multiple factors and variables and has been demonstrated as a useful approach to solve multiple-attribute decision-making problems [31]. Generally speaking, one of the main purposes of relation analysis is to identify the importance order of factors, so the GRD rankings are more significant [32]. Overall, GRA, PCA, RR, and RBF network were combined to control the mutual interference among the 21 component variables to better assess the data.

### 6 The core bioactive components promoting blood circulation

Under the hypothesis that the effects of CXC samples varied with component ratio, the relationship between component and effect differences was analyzed to evaluate the contribution of each component using the methods of GRA, PCA, and RR-RBF. The computed results are shown in Table 8. In addition, a dynamic component-effect analysis bubble chart based on the analysis results is provided as Component-effect relevance bubble chart S1 to provide a clear picture of the comprehensive effects of all 21 components.

**Table 8:** The relevance results between 21 components and 5 factors.

	F1				F2				F3				F4				F5			
	GRD	PCA RC10^−2^	RR SRRC	RBF BV/SIV	GRD	PCC	RR SRRC	RBF BV/SIV	GRD	PCC	RR SRRC	RBF BV/SIV	GRD	PCC	RR SRRC	RBF BV/SIV	GRD	PCA RC10^−2^	RR SRRC	RBF BV/SIV
P1	0.64	−2.30			0.67	−0.08	0.51	1.12/88.0%	0.60	−0.22	−0.61	−4.18/100%	0.64	−0.04			0.83	4.57	0.62	10.21/100%
P2	0.54	−5.79			0.64	−0.01	−0.2	−0.04/92.5%	0.59	−0.01	0.16	0.40/51.0%	0.62	−0.41			0.67	1.25		
P3	0.68	0.14			0.74	0.34	0.24	3.28/89.7%	0.64	0.05	0.14	2.97/91.4%	0.65	−0.22	−0.52	−5.10/94.6%	0.67	−4.18		
P4	0.72	1.89	0.16	5.63/40.3%	0.73	−0.26			0.64	0.13	0.25	1.26/50.5%	0.69	0.21			0.72	3.18		
P5	0.53	−5.83			0.62	0.00	−0.21	−0.47/100%	0.56	−0.07			0.62	−0.37			0.65	1.33		
P6	0.73	2.23	0.2	5.71/37.6%	0.73	−0.19			0.64	0.10	0.3	0.85/52.9%	0.69	0.21			0.72	2.99		
P7	0.73	2.46			0.74	−0.22			0.64	0.11	0.08	0.51/48.4%	0.69	0.21			0.72	2.84		
P8	0.53	−5.82			0.63	0.01			0.57	−0.05			0.61	−0.40	−0.16	−2.27/90.7%	0.66	1.30		
P9	0.62	−1.71	−0.17	−0.74/41.4%	0.68	−0.23			0.61	−0.05			0.60	0.03			0.79	4.64		
P10	0.74	2.66	0.13	5.74/37.5%	0.74	−0.18			0.64	0.10	0.25	1.05/49.7%	0.69	0.23			0.72	2.69		
P11	0.72	2.36			0.73	−0.27			0.64	0.11	0.15	−0.04/49.0%	0.69	0.27	0.51	6.89/100%	0.72	2.88		
P12	0.56	−5.77			0.65	0.02			0.59	0.01	0.27	1.80/59.3%	0.64	−0.38			0.66	1.39		
P13	0.60	−4.84			0.74	0.07			0.66	0.08	0.34	2.78/48.6%	0.63	−0.37			0.73	−0.47		
P14	0.53	−5.81	−0.26	−7.75/57.7%	0.62	0.02			0.57	−0.06			0.61	−0.39			0.65	1.28		
P15	0.62	−1.68			0.65	−0.24			0.62	−0.04			0.58	−0.07	−0.42	−5.36/84.3%	0.75	4.64		
P16	0.61	−1.90	−0.2	−1.56/38.7%	0.62	−0.31	−0.18	−1.35/93.0%	0.58	−0.06			0.59	0.04			0.70	4.64		
P17	0.67	2.06	0.1	0.87/36.4%	0.72	0.31	0.19	2.19/80.7%	0.63	0.08	0.12	2.88/86.0%	0.68	−0.04			0.61	−4.66		
P18	0.63	−2.07			0.63	−0.20			0.60	−0.02			0.60	−0.03	−0.15	−1.77/89.2%	0.73	4.70		
P19	0.73	3.63	0.26	10.21/100%	0.72	−0.42	−0.52	−6.36/99.5%	0.65	0.29	0.23	0.42/50.1%	0.71	0.16			0.70	1.09	0.14	6.03/61.4%
P20	0.55	−5.53			0.65	−0.02			0.60	−0.02			0.63	−0.32			0.67	0.89	0.33	0.33/48.2%
P21	0.54	−5.83			0.63	0.02			0.58	−0.07			0.63	−0.38			0.67	1.37	0.16	0.87/41.9%

a) For GRD, RC, PCC, SRRC and BV, positive and negative of value means positive and negative effect contribution respectively. The larger the absolute value, the higher the effect contribution. SIV represents the relative importance and reflects the reliability of corresponding variables.

b) After comprehensive comparison, P19, P10, P6, and P4 made the main effect contribution to RBC aggregation (F1); P3 and P17 to platelet aggregation (F2); P13, P12, P10, and P19 to intrinsic clotting activity (F3); P11 to RBC deformability and plasma proteins (F4); P1 to extrinsic clotting activity (F5). Meanwhile, P14, P19, P1, and P15 might have negative effect on F1, F2, F3, and F4, respectively.

c) P19 (panaxytriol), P10 (ginsenoside Rb_1_), P3 (angoroside C), P13 (protocatechualdehyde), P11 (ginsenoside Rd), and P1 (calycosin-7-O-β-D-glucoside) are the core bioactive components promoting blood circulation. P14 is rosmarinic acid. P15 is ononin.

F1 represents RBC aggregation in the vascular system. The results show that panaxytriol and ginsenoside Rb_1_ were the major effective components to suppress RBC aggregation. Ginsenoside Rb_1_, with high PN saponin content, can ameliorate lipopolysaccharide (LPS)-induced microcirculatory disturbance in the rat mesentery [33]. The ability of ginsenoside Rb_1_ to suppress RBC aggregation, revealed in this study, might be the foundation of its effects on blood circulatory disturbances, as evidenced by increasing studies. Panaxytriol has a relatively low content in PN and has only been reported to exhibit potent anti-tumor properties [34]. Until now, there have been no descriptions of the pharmacological effects of panaxytriol against vascular dysfunction. The results of this study suggest that it could be useful to further investigate panaxytriol's novel therapeutic effects.

F2 represents platelet aggregation in the vascular system. Angoroside C was more closely related to platelet aggregation than the other components. Angoroside C is one of the main compounds involved in the anti-inflammatory effect of RS and has a significant effect on thromboxane B_2_ (TXB_2_) release and nitric oxide (NO) activity in LPS-induced macrophages [35]. Thromboxane A_2_ (TXA_2_) has been demonstrated to induce platelet aggregation, and TXB_2_ is the stable hydrolysate of TXA_2_. Therefore, angoroside C's significant suppression of platelet aggregation might be partly attributed to the effect on TXB_2_ release, which ameliorates blood hypercoagulability.

F3 represents intrinsic clotting activity in the vascular system. All 21 components exhibited relatively low relevance to F3, suggesting that the contribution of every single component to F3 was small. Nevertheless, the results showed that protocatechualdehyde had the closest relevance to F3 and might be the bioactive component. Protocatechualdehyde is known for its antiproliferative and antioxidant activities, as well as its ability to inhibit aldose reductase in rat lens epithelial cells [36]. This study indicated that protocatechualdehyde might influence intrinsic clotting activity and could be of therapeutic interest with respect to promoting blood circulation.

F4 represents RBC deformability and plasma proteins in the vascular system, which is closely related to blood microcirculatory disturbance. In this study, ginsenoside Rd had a significantly higher influence on F4 than the other components. More than 20 basic research papers on ginsenoside Rd have been recently published in many journals. In these studies, ginsenoside Rd was reported to have many different effects as a bioactive component: it could attenuate early oxidative damage and sequential inflammatory responses after transient focal ischemia in rats [37], relieve redox imbalance and improve stroke outcome after focal cerebral ischemia in aged mice [38], and prevent the development of atherosclerosis by blocking Ca^2+^ influx through receptor- and store-operated Ca^2+^ channels in vascular smooth muscle cells [39], [40]. The present study provides evidence for ginsenoside Rd's possible regulation of RBC deformability and plasma proteins, yet elucidation of the underlying mechanism requires further research.

F5 represents extrinsic clotting activity in the vascular system. We found that calycosin-7-O-β-D-glucoside was most closely related to F5. It has been reported that calycosin-7-O-β-D-glucoside can increase endogenous antioxidant levels [41] and improve cell membrane fluidity in the brains of rats subjected to experimental stroke [42]. However, there is still no direct evidence showing calycosin-7-O-β-D-glucoside's activity on the extrinsic clotting pathway. The present study indicates a new potential pharmacological activity of calycosin-7-O-β-D-glucoside.

We determined that panaxytriol, ginsenoside Rb_1_, angoroside C, protocatechualdehyde, ginsenoside Rd, and calycosin-7-O-β-D-glucoside were the core bioactive components of CXC and that they acted on different aspects of the vascular system. Panaxytriol and ginsenoside Rb_1_ were relevant to RBC aggregation, angoroside C to platelet aggregation, protocatechualdehyde to intrinsic clotting activity, ginsenoside Rd to RBC deformability and plasma proteins, and calycosin-7-O-β-D-glucoside to extrinsic clotting activity. Moreover, this study provides an important reference for the development of novel therapeutics on promoting blood circulation that employ angoroside C, calycosin-7-O-β-D-glucoside, panaxytriol, and protocatechualdehyde.

Besides, according to the attribution of 6 bioactive components, PN might affect two aspects of the vascular system, including F1 (RBC aggregation) and F4 (RBC deformability and plasma proteins), while RA, RS, and SM might influence the F5 (extrinsic coagulation system), F2 (platelet aggregation), and F3 (intrinsic coagulation activity), respectively. Consequently, this work also partly revealed the mutual promotion among 4 herbs and demonstrated the multicomponent and multitarget properties of CXC.

However, the results also showed that rosmarinic acid, ononin, calycosin-7-O-β-D-glucoside, and panaxytriol might have negative effects on RBC aggregation, RBC deformability, intrinsic clotting activity, and platelet aggregation (Table 8). Both CHFs and organisms are very complex systems and the specific interactions between them are still unclear; therefore, further exploration and verification are needed to understand the pharmacological mechanisms of the 4 components listed above. Nevertheless, this study demonstrated that one specific aspect of the vascular system might be affected by two or more CXC components, and the same component might have a positive effect on one aspect yet a negative effect on another, which reflects both the synergistic and the antagonistic actions of CHF components.

**Figure 6:**
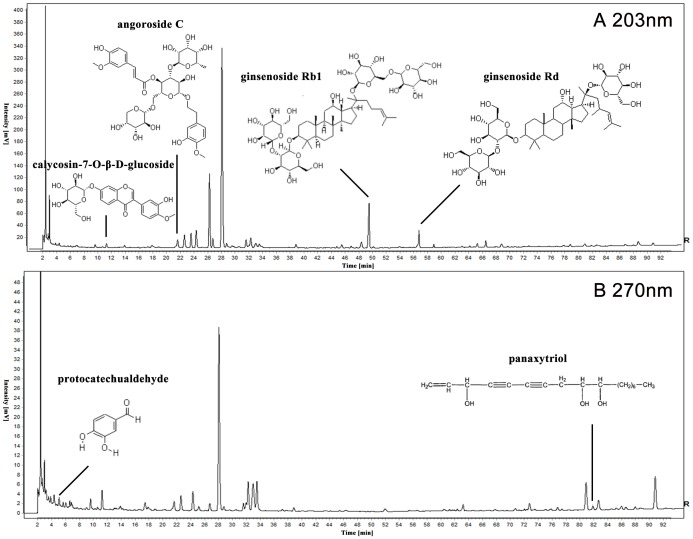
The standard bioactive HPLC fingerprint of CXC. A, B respectively represents the standard HPLC fingerprint of CXC at wavelength 203 nm and 270 nm. The core bioactive components are all displayed in the standard fingerprint for qualitative or quantitative detection.

Comprehensive qualitative or quantitative detection based on the low or high content of core bioactive components is necessary to improve CXC quality control. Therefore, we established the bioactive HPLC fingerprint of CXC based on its core bioactive components (Fig. 6).

In summary, we explored the core bioactive components of CXC with a series of HPLC and animal experiments. The results suggested that the core bioactive components of CXC promoting blood circulation were panaxytriol, ginsenoside Rb_1_, angoroside C, protocatechualdehyde, ginsenoside Rd, and calycosin-7-O-β-D-glucoside. Overall, these findings provide a novel perspective to pinpoint the core bioactive components in a CHF, which will be helpful for improving quality control and inspiring further clinical studies of CHFs. It might also provide guidance for drug discovery.

## Supporting Information

Table S1 **The results of the animal experiments in 13 treatment groups.** Control group and model group received the same volume of normal saline (NS) for the treatment. ^*^ *P*<0.05 and ^**^ *P*<0.01 vs control group, ^#^ *P*<0.05 and ^##^ *P*<0.01 vs model group, n = 10.
(DOCX)

Component-Effect Relevance Bubble Chart S1 **A dynamic bubble chart based on the component-effect analysis results.** The relevance between 21 components and 5 factors (F1–F5) is shown to provide a clear picture of comprehensive effects of all 21 components.
(SWF)
