# Supplementary material for: Core Bioactive Components Promoting Blood Circulation in the Traditional Chinese Medicine Compound Xueshuantong Capsule (CXC) Based on the Relevance Analysis between Chemical HPLC Fingerprint and In Vivo Biological Effects
Source: PLoS One. 2014 Nov 14;9(11):e112675. doi: 10.1371/journal.pone.0112675 (PMC4232446; doi:10.1371/journal.pone.0112675)
Supplement: Table S1 — The results of the animal experiments in 13 treatment groups. Control group and model group received the same volume of normal saline (NS) for the treatment. * P<0.05 and ** P<0.01 vs control group, # P<0.05 and ## P<0.01 vs model group, n = 10. (DOCX) [file pone.0112675.s001.docx]

**Table S1.** The results of the animal experiments in 13 treatment groups

| Group | Dose (mg/kg/d) | WBV (mPa.s) | | | | | PT (s) | APTT (s) | PV120/s  (mPa.s) | MPAR | EAI | RCEI |
| --- | --- | --- | --- | --- | --- | --- | --- | --- | --- | --- | --- | --- |
|  |  | 5/s | 30/s | 50/s | 150/s | 200/s |  |  |  |  |  |  |
| Control | NS | 7.96±0.54 | 6.18±0.46 | 5.58±0.37 | 4.78±0.29 | 4.49±0.17 | 8.74±0.28 | 14.04±1.03 | 0.99±0.02 | 33.33±4.20 | 1.72±0.12 | 4.87±0.23 |
| Model | NS | 10.00±1.14^**^ | 7.52±0.80^*^ | 6.75±0.66^*^ | 5.32±0.38^*^ | 4.92±0.34^*^ | 7.98±0.25^**^ | 12.29±0.93^*^ | 1.17±0.02^**^ | 40.33±3.24^*^ | 2.12±0.18^**^ | 3.69±0.31^**^ |
| Asp | 100 | 7.58±1.59^##^ | 6.28±0.93^##^ | 5.73±0.72^##^ | 4.96±0.38^#^ | 4.86±0.39 | 8.13±0.30 | 13.01±0.70 | 1.16±0.01 | 31.47±6.19^##^ | 1.58±0.26^##^ | 4.04±0.89 |
| CDDP | 800 | 7.34±0.83^##^ | 6.03±0.39^##^ | 5.54±0.34^##^ | 4.87±0.18^##^ | 4.80±0.17 | 8.10±0.29 | 12.81±1.09 | 1.16±0.04 | 36.41±3.21 | 1.62±0.29^##^ | 3.89±0.77 |
| S1 | 1520 | 8.50±1.25^#^ | 6.15±0.55^##^ | 5.64±0.46^##^ | 4.94±0.27^##^ | 4.73±0.34 | 8.00±0.18 | 13.26±1.36 | 1.16±0.03 | 33.10±2.90^##^ | 1.65±0.22^##^ | 4.11±0.30 |
| S2 | 1520 | 7.38±0.78^##^ | 6.03±0.40^##^ | 5.53±0.36^##^ | 4.90±0.30^##^ | 4.85±0.26 | 7.98±0.25 | 13.19±0.81 | 1.17±0.04 | 37.97±1.15 | 1.56±0.17^##^ | 4.25±0.28^#^ |
| S3 | 1520 | 8.49±1.33^#^ | 6.23±0.23^##^ | 5.70±0.16^##^ | 4.95±0.08^#^ | 4.90±0.09 | 8.14±0.34 | 13.79±1.22^#^ | 1.16±0.03 | 39.32±2.86 | 1.67±0.13^##^ | 3.67±0.12 |
| S4 | 1520 | 7.66±0.86^##^ | 6.18±0.30^##^ | 5.71±0.24^##^ | 5.00±0.22^#^ | 4.95±0.17 | 8.04±0.23 | 13.60±0.92 | 1.18±0.02 | 36.54±4.82 | 1.53±0.16^##^ | 4.35±0.44^#^ |
| S5 | 1520 | 7.53±1.09^##^ | 6.19±0.32^##^ | 5.68±0.33^##^ | 4.92±0.17^##^ | 4.83±0.20 | 8.08±0.36 | 13.84±1.20^#^ | 1.15±0.02 | 30.95±5.78^##^ | 1.61±0.22^##^ | 4.62±0.24^##^ |
| S6 | 1520 | 9.28±1.69 | 7.00±0.51 | 6.38±0.34 | 5.24±0.20 | 4.98±0.16 | 8.10±0.30 | 12.93±1.51 | 1.19±0.03 | 38.16±2.99 | 1.87±0.30^#^ | 3.64±0.25 |
| S7 | 1520 | 9.22±1.57 | 6.73±0.72^##^ | 6.40±0.64 | 5.22±0.38 | 5.06±0.37 | 7.88±0.16 | 13.45±1.37 | 1.17±0.03 | 40.48±2.18 | 1.94±0.35 | 3.62±0.23 |
| S8 | 1520 | 8.82±1.23 | 6.66±0.36^##^ | 6.07±0.27^##^ | 5.22±0.20 | 4.88±0.14 | 8.06±0.29 | 12.70±1.36 | 1.17±0.03 | 37.52±3.16 | 1.83±0.21^#^ | 3.57±0.32 |
| S9 | 1520 | 10.11±0.93 | 7.25±0.24 | 6.40±0.18 | 5.38±0.14 | 5.12±0.21 | 8.06±0.42 | 13.15±0.48 | 1.18±0.04 | 36.53±1.27 | 2.00±0.23 | 3.71±0.42 |

a) Control group and model group received the same volume of normal saline (NS) for the treatment.

b) ^*^ *P<*0.05 and ^**^ *P<*0.01 vs control group, ^#^ *P<*0.05 and ^##^ *P<*0.01 vs model group, n=10.
